# Effectiveness of acidic deep eutectic solvents in recovery of hazardous base metals from waste printed circuit boards

**DOI:** 10.1007/s11356-025-36685-w

**Published:** 2025-06-26

**Authors:** Emmanuel Anuoluwapo Oke, Johannes Herman Potgieter

**Affiliations:** Sustainable and Innovative Minerals and Metals Extraction Technology (SIMMET) Research Group, School of Chemical and Metallurgical Engineering, University of the Witwatersrand, Private Bag X3 PO Wits 2050, Johannesburg, South Africa

**Keywords:** Electronic waste, Environmental protection, Green solvents, Thermogravimetric analysis, Activation energy

## Abstract

Traditional methods for metal recovery from printed circuit boards (PCBs) are often associated with high costs, toxicity, and environmental risks. This study explores the use of acidic deep eutectic solvents (DESs) as a green alternative for recovering Pb, Cr, Zn, and Ni from waste PCBs. Three DESs were prepared using choline chloride (ChCl) as a hydrogen bond acceptor (HBA) and paired with acetic acid (AA), chloroacetic acid (CAA), or dichloroacetic acid (DCA) as hydrogen bond donors (HBDs). The effect of DES nature on the recovery of the investigated metals follows the sequence ChCl:DCA > ChCl:CAA > ChCl:AA. The results revealed that the ChCl:DCA DES exhibited the highest recovery efficiency, achieving 89.5% for Pb, 55.2% for Cr, 80.5% for Zn, and 88.6% for Ni at 50 °C for 3 h in the presence of 1.0 M H_2_O_2_ and a stirring speed of 500.0 rpm. In addition, recovery efficiencies of 99.8%, 71.8%, 100.0%, and 84.9% were achieved for Pb, Cr, Zn, and Ni, respectively, when 40.0 wt% water was added to the ChCl:DCA DES. The shrinking core model (SCM) reveals that the recovery of Pb, Cr, Zn, and Ni from waste PCBs is governed by a diffusion-controlled mechanism. The activation energies were determined to be 19.8 kJ/mol for Pb, 32.4 kJ/mol for Cr, 14.3 kJ/mol for Zn, and 30.2 kJ/mol for Ni. This study offers a promising and highly sustainable alternative for the recovery of hazardous metals from waste PCBs, contributing to a benign environmental approach and process.

## Introduction

Owing to the increasing world population and organisations acquiring electrical and electronic devices, waste electrical and electronic equipment (WEEE) has become one of the largest waste streams globally by volume. Exports of WEEE from developed to developing nations often result in an increase in the overall amount of electronic waste (e-waste) in the destination countries (Holuszko et al. 2022). An often overlooked aspect of WEEE is hazardous waste, generated by products containing Pb, Hg, Cr, Ni, As, Cd, Li, and other potentially toxic elements (Przydatek and Kanownik 2024). A serious risk of human exposure and environmental contamination exists in developing nations due to improper and inadequate handling of e-waste (Preston-Whyte and Maes 2023). Toxic metals and compounds in e-waste that are improperly managed can pose major risks to human health and the environment. When e-waste is burned, toxic compounds are released into the air, causing respiratory issues, as well as other health problems (Oke and Potgieter 2024a, b). In addition, they can contaminate water and soil, damaging food sources and the ecosystem (Mouton and Roux 2024).

Pb, Zn, Ni, and Cr, among others, are commonly occurring toxic metals found in a wide range of electronic products. These hazardous metals are found in trace amounts in numerous electronic components, including printed circuit boards (PCBs), batteries, resistors, and capacitors (Oke et al. 2023; Parvez et al. 2024; Oke and Potgieter 2024a). Pb can have serious detrimental effects on the body, particularly targeting the kidneys, reproductive system, and nervous system (Jain et al. 2023). Prolonged exposure to Pb may result in damage to these organs, potentially leading to severe health conditions. Pb can also cause blood disorders and impair brain function, contributing to cognitive deficits, developmental delays in children, and even neurological diseases in adults (Zeng et al. 2020; Desye et al. 2023). Furthermore, its accumulation in the body over time can exacerbate these effects, posing a long-term threat to human health. Furthermore, exposure to Ni can result in a range of negative health effects, including allergic reactions, kidney and heart issues, lung fibrosis, and an elevated risk of lung and nasal cancer (Iqbal Khan et al. 2023). Although the exact molecular mechanisms behind Ni toxicity are not yet fully understood, it is believed that mitochondrial dysfunction and oxidative stress play a vital and central role in this metal’s toxicity (Ugulu 2015). In addition, chronic exposure to Cr can lead to severe health issues, including burns, anaemia, allergic reactions, and damage to the small intestine and stomach. It can also negatively impact the male reproductive system and sperm and is associated with cancers of the kidney, liver, and lungs, as well as stomach damage and skin irritation (Hossini et al. 2022). In addition to its effects on human health, Cr is detrimental to plants, impairing their development by affecting germination, root growth, and overall plant health, which can decrease crop yield and total dry matter production (Koc et al. 2024).

In their 2017 study, Feifan and Mengjun achieved less than 10.0% leaching efficiency for Pb from waste PCBs using 80.0% N-sulfobutylpyridinium trifluoromethanesulfonate ionic liquid (IL) and 25.0% H_2_O_2_ (Feifan and Mengjun 2017). This process demonstrated disappointingly low Pb leaching efficiency. Also, in their 2021 study, Sabzkoohi and Kolliopoulos investigated Pb and Ni leaching from waste PCBs using a deep eutectic solvent (DES) composed of ChCl and ethylene glycol with 0.1 M I_2_. Despite conducting the process at 85.0 °C, with a duration of 72.0 h and stirring at 150 rpm, the leaching efficiency obtained for Pb was less than 10%. In contrast, about 75.0% leaching efficiency was achieved for Ni. Furthermore, in their recent study, Anwer et al. achieved 94.0% Pb leaching efficiency from waste PCBs using 0.5 M citric acid and 5.8% H_2_O_2_ (Anwer et al. 2022). The process was carried out at 30.0 °C, with a particle size of 7.0 × 7.0 mm, stirring at 150 rpm, and using a leaching time of 4 h. The leaching of 90.0% Ni from waste PCBs in an ammonia-ammonium solution with the addition of 0.4 M H_2_O_2_ has been reported as well (Jadhao et al. 2021). Additionally, Zn leaching from PCBs using the ammonia-ammonium sulphate system achieved a maximum recovery of 94.0% after 30 h when H_2_O_2_ was used as the oxidant (Pinho et al. 2021). The optimal conditions included a stirring rate of 500 rpm, an L/S ratio of 40, and twice the stoichiometric amount of ammonia. The bioleaching performance of *Acidithiobacillus ferrooxidans* for Pb and Cr, even after mechanical activation, remained relatively low (Gu et al. 2019). Specifically, the leaching efficiency was only 10.0% for Pb and 75.0% for Cr, indicating limited effectiveness of the process. In another study, the bioleaching of Ni from pre-treated waste PCBs using *Aspergillus niveus* achieved a maximum recovery of 73.6% (Krishnamoorthy et al. 2021). This recovery was accomplished over 15 days with particle sizes of 60–80 mesh, utilising itaconic and oxalic acids produced by the fungus, while the citric acid titre decreased in both one-step and two-step bioleaching methods.

Because ammonia-ammonium solutions are volatile or corrosive by nature, using them for leaching raises serious safety issues, especially when done at high temperatures. On the other hand, secondary environmental contamination can result from these leaching methods (Oke and Potgieter 2024a, b). The use of ILs and DESs for the recovery of hazardous metals from waste PCBs is challenged by low extraction efficiencies, as demonstrated in some studies. Additionally, ILs face the drawbacks of high costs and potential environmental concerns due to their toxicity and limited biodegradability. DESs, on the other hand, are more affordable and less toxic. In addition, bioleaching has a major drawback that, while recovering hazardous metals from waste PCBs, it has a relatively slow processing time compared to chemical methods. Bioleaching often requires extended periods for microorganisms to produce enough acids and achieve significant metal recovery, which can be a limitation for large-scale operations (Xia and Ghahreman 2024). Additionally, the efficiency of bioleaching can be affected by the type of microorganisms and other factors (Yaashikaa et al. 2022; Ji et al. 2022). Owing to the bottlenecks associated with the existing methods, it is germane to develop fast, efficient, and less environmentally toxic methods for recovering toxic base metals from waste PCBs. Because of their many benefits, which primarily include flexible designability, property tunability, and high biodegradability, DESs, a type of green solvent that was developed recently, have drawn more and more attention (Oke 2024b, a). They are frequently employed in metal recovery and separation due to their high solubility with metal materials (Guo et al. 2024). More and more research has recently documented their use in the sustainable recovery of metals from electronic waste, primarily from materials from spent batteries, PCBs, lamp phosphors, and end-of-life permanent magnets (Oke and Potgieter 2024b; Guo et al. 2024).

Not only can DESs improve the efficiency of the separation and extraction process, but they are also a compelling reason for investigating metal recovery based on DESs. Therefore, in this present study, deep eutectic solvents (DESs) are explored as a potential replacement for toxic inorganic solvents, other hazardous lixiviants, and bioleaching while decontaminating PCBs before disposal into the environment. So, a simple, fast, and environmentally benign method for recovering hazardous base metals (Pb, Zn, Cr, and Ni) from waste PCBs has been developed through the use of choline chloride (ChCl) as hydrogen bond acceptor (HBA) in three different DESs formulations. Each formulation pairs acetic acid (AA), chloroacetic acid (CAA), or dichloroacetic acid (DCA) as hydrogen bond acceptors (HBDs). No study has reported the efficient recovery of hazardous metals from waste PCBs using DESs composed of ChCl, AA, CAA, and DCA. The selection of ChCl as the HBA in the DESs formulations is based on its ability to readily form hydrogen bonds, its low toxicity, biodegradability, and affordability (Mishra et al. 2023). ChCl is a quaternary ammonium salt with a strong affinity for HBDs, facilitating the formation of stable DESs. Also, AA, CAA, and DCA were chosen as HBDs due to their varying acid strengths and electron-withdrawing properties (Cui et al. 2017). AA is a weak organic acid that offers moderate acidity, while the substitution of chlorine atoms in CAA and DCA increases the acidity and polarizability of the solvents, enhancing their ability to interact with metal ions. These properties make the DESs highly effective for solubilising and extracting hazardous metals such as Pb, Zn, Cr, and Ni from complex waste matrices like PCBs. Additionally, introducing water into the DES system before metal recovery, is a novel approach, further enhances the recovery efficiency, contributing to both e-waste management and environmental sustainability.

## Materials and experimental methods

### Chemicals

All chemicals used in this study were of reagent grade, sourced from Sigma-Aldrich (USA) and Associated Chemical Enterprises Pty Ltd (South Africa). Unless otherwise specified, all chemicals and reagents were used as supplied, without any additional purification or treatment. Detailed information on the chemicals utilised in this study is found in Table 1.

**Table 1:** Detailed information on all chemicals used in this study

Chemicals	Acronym	Molecular weight (g/mol)	Sources	CAS no	Purity (%)
Choline chloride	ChCl	139.62	Sigma-Aldrich	67–48-1	≥ 99.0%
Acetic acid	AA	60.05	Sigma-Aldrich	64–19-7	≥ 99.8%
Chloroacetic acid	CAA	94.49	Associated Chemical Enterprises Pty Ltd	79–11-8	≥ 99.0%
Dichloroacetic acid	DCA	128.94	Sigma-Aldrich	79–43-6	≥ 99.0%
Hydrogen peroxide	H_2_O_2_	34.00	Associated Chemical Enterprises Pty Ltd	2877–00-00	50.0 %
Iodine	I_2_	253.81	Sigma-Aldrich	7553–56-2	≥ 99.8%
Potassium permanganate	KMnO_4_	158.03	Sigma-Aldrich	7722–64-7	≥ 99.0%
Ethanol	ETOH	46.07	Sigma-Aldrich	64–17-5	≥ 99.5%

### Preparation of waste PCBs

Using the available tools, PCBs from discarded desktop computers (HP brand) were disassembled by manually recovering large components that could not be crushed, like connectors for peripheral devices and stainless-steel heat sinks. Using a saw, the disassembled PCBs were cut into smaller pieces.

### Epoxy layer removal from waste PCBs

**Fig. 1:**
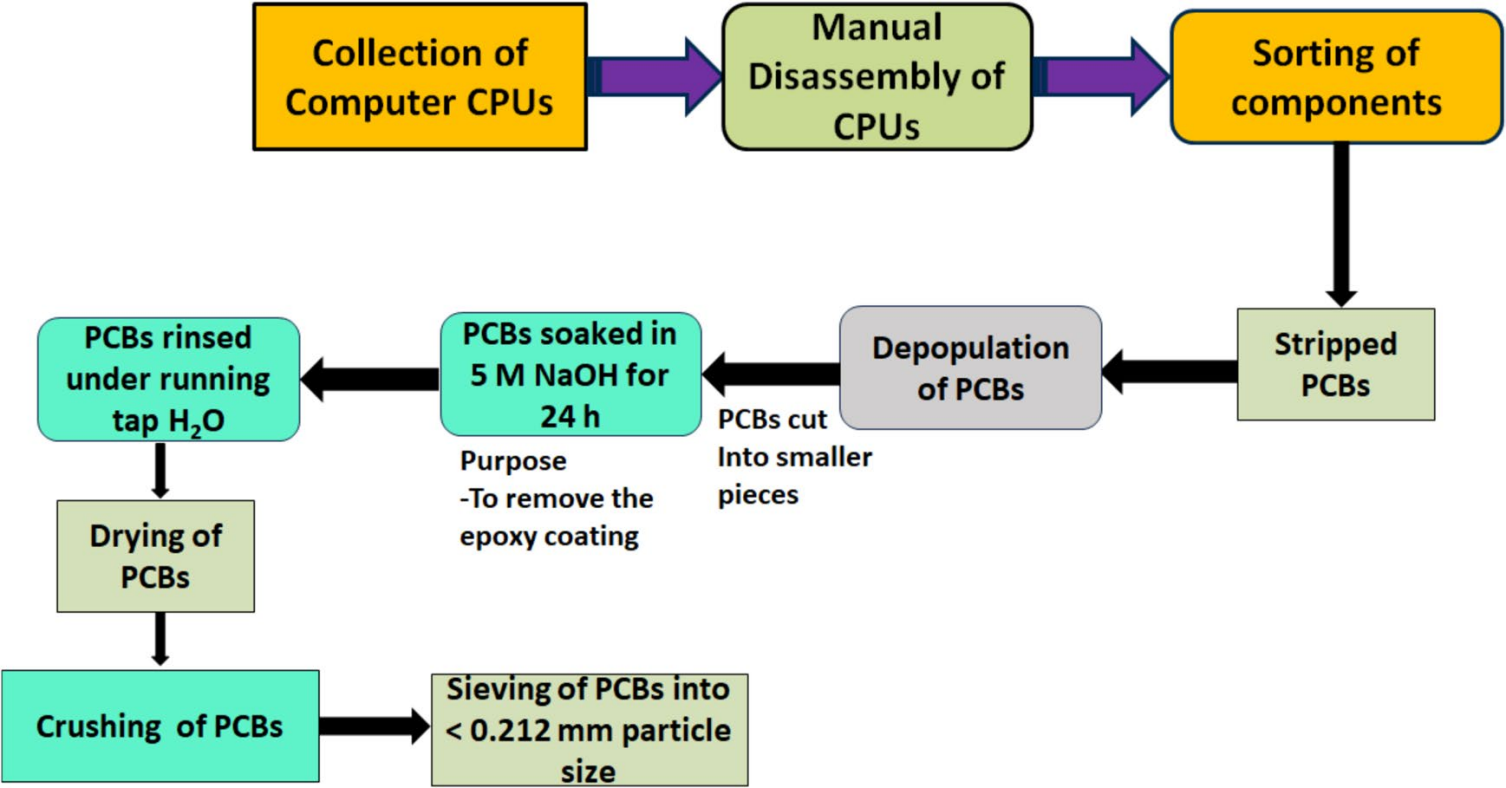
Sample collection and preparation flowsheet

The epoxy layer, also referred to as a solder mask, is an organic green layer that covers the top of PCBs and serves as a protective layer for the metal components. It also resists heat and corrosion and shields the PCB from the environment. On PCBs, various epoxy coatings are applied, and each PCB may have a unique composition. Nevertheless, the epoxy coating of the PCBs must be removed. It hinders the dissolution of the metal and prevents the leaching agent from penetrating the metal to dissolve it (Anwer et al. 2022). After experimenting with several methods, it was observed that 5.0 M NaOH works well to remove the epoxy layer (Nagarajan and Panchatcharam 2023). After being immersed in 5.0 M NaOH for 24.0 h, the disassembled PCB was rinsed with deionised water to get rid of any remaining NaOH residue. The use of 5 M NaOH in this study was confined to a standard pre-treatment step, aimed at removing the epoxy resin layer from waste PCBs before leaching. This method is commonly employed across various hydrometallurgical processes, irrespective of the leaching solvent applied, and is not unique to DES-based systems. Although NaOH is a strong base and corrosive, its application in this context is well-established and carried out under controlled laboratory conditions with proper waste management protocols. As such, its limited and conventional use in this preliminary stage does not significantly detract from the overall sustainability of the process. After being treated with NaOH, the metallic layer became fully visible, and the epoxy coating was completely removed. The treated PCBs were thoroughly dried before being crushed and sieved through a sieve of mesh size of 0.212 mm to liberate metals from the non-metallic resin of the board and to increase the surface area exposed to the leaching agent. All the processes involved from the sample collection to the sieving of crushed PCBs are illustrated in Fig. 1.

### Characterisation of ground waste PCBs

The ground sample of waste PCBs was subjected to characterisation using X-ray fluorescence (XRF) and scanning electron microscopy (SEM) before the recovery experiments. XRF was utilised to determine the elemental composition of the waste PCB. The powdered sample was pressed into a pellet and analysed using an XRF spectrometer, and the results obtained for the main hazardous metals found in the waste PCB sample are presented in Table 2. In addition, the surface morphology and elemental composition of the waste PCB sample were further analysed using SEM. The powdered sample was mounted on carbon tape and sputter-coated with a thin layer of gold to ensure conductivity. SEM imaging was conducted under a high vacuum at an accelerating voltage of 10.0 kV to observe the microstructural features.

**Table 2:** XRF results of hazardous metals in waste PCBs sample before the recovery process

Ni (mass %)	Zn (mass %)	Pb (mass %)	Cr (mass %)
1.248	0.442	1.089	0.316

### Preparation and Fourier-transform infrared (FT-IR) spectroscopy characterisation of acidic DESs

Three DESs constituted by ChCl (HBA) and AA, CAA, and DCA as HBDs (i.e. ChCl:AA, ChCl:CAA, and ChCl:DCA) were prepared in a 1:2 molar ratio. The mixtures were stirred using a thermostatically controlled hot plate equipped with a magnetic stirrer set at 40 °C and 500 rpm until a homogeneous solution was obtained without any precipitates. This is similar to the method employed by Ferrera et al. in their recent study on the stability of ChCl DESs based on carboxylic acids (Ferreira et al. 2024). Once the DESs were formed, they were placed in a desiccator to remove any moisture. The DESs obtained were stored in tightly sealed glass vials to safeguard them from structural alterations or external environmental influences on their physical and chemical characteristics. The DESs were ready for use without requiring any additional purification. Also, the prepared DESs were characterised by Fourier-transform infrared (FT-IR) spectroscopy to analyse their chemical structures.

#### Thermogravimetric (TGA) analysis

A TGA (SDT-Q600, TA Instruments) was run in a nitrogen atmosphere with a heating rate of 10 °C/min and a temperature range of 20–340 °C. TGA has a 0.01% weighing precision and an isothermal temperature precision. This has been used to assess spot phase changes brought on by oxidation, dewatering, or degradation as well as the heat stability (represented by a weight variation) of DES constituted by ChCl and DCA (ChCl:DCA). Three replicate calibration runs were utilised to ascertain the relative temperature accuracy using the nickel and alumel Curie point standards.

### Recovery procedure

**Fig. 2:**
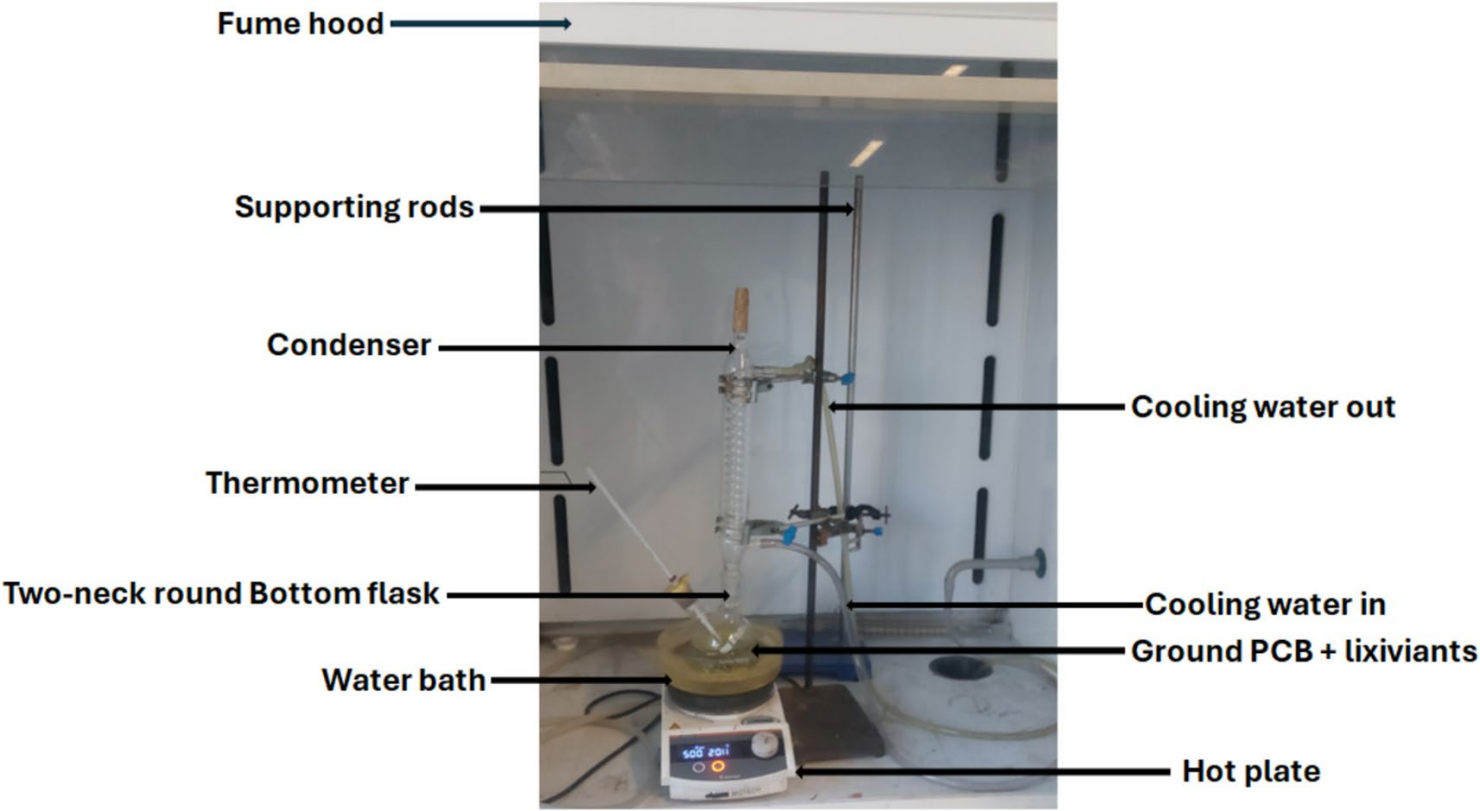
Detailed leaching setup used in this work

The leaching setup is presented in Fig. 2. In this study, a 1:10 solid-to-liquid (S/L) ratio was used. The recovery experiment for the ground waste PCB sample in the DES with the H_2_O_2_ oxidant (it should be noted that 10% of 1 M H_2_O_2_ was added to 90% DES during the leaching procedure whenever it is applicable) was conducted under atmospheric conditions in a 250-mL two-necked round-bottom flask. The flask was immersed in a water bath and placed on a thermostatically controlled hot plate equipped with a magnetic stirrer. One neck of the flask, acting as the leaching reactor, was connected to a water-cooled condenser, while the other held a thermometer submerged in the leaching solution. A stirring bar inside the flask ensured uniform mixing at 500 rpm. The recovery temperatures varied between 30 and 80 °C, and the duration which ranged from 1 to 6 h was investigated. The resulting solution was filtered using a microfibre filter with 0.45 µm pore size and diluted with deionised water before the metals of interest were analysed using the inductively coupled plasma mass spectrometer (ICP-MS). To reduce potential errors, all experiments were performed in duplicate. The percentage of metal leached was estimated using Eq. (1).

1 $$\text{Recovery efficiency }\left({\%}\right)=\frac{C \times V}{M \times Z} \times 100$$

In this case, *C* denotes the concentration of the target metal in the recovered solution, *V* represents the volume of the recovered solution, *M* denotes the amount of metal of interest within the original sample, and *Z* depicts the weight of the sample.

In this leaching stage, 10% of 1 M H_2_O_2_ was added to the 90% DES to enhance metal dissolution as previously stated. The use of H_2_O_2_ in this context aligns well with green chemistry principles, as H_2_O_2_ is recognised as an environmentally benign oxidant. It decomposes into water and oxygen, producing no toxic byproducts (Ciriminna et al. 2016; Poursaitidis et al. 2024). The moderate concentration and small volume employed in this study further minimise any environmental impact, especially when compared with conventional leaching agents such as aqua regia, cyanide, and other toxic solvents.

## Results and discussion

### DESs FT-IR characterisation

**Fig. 3:**
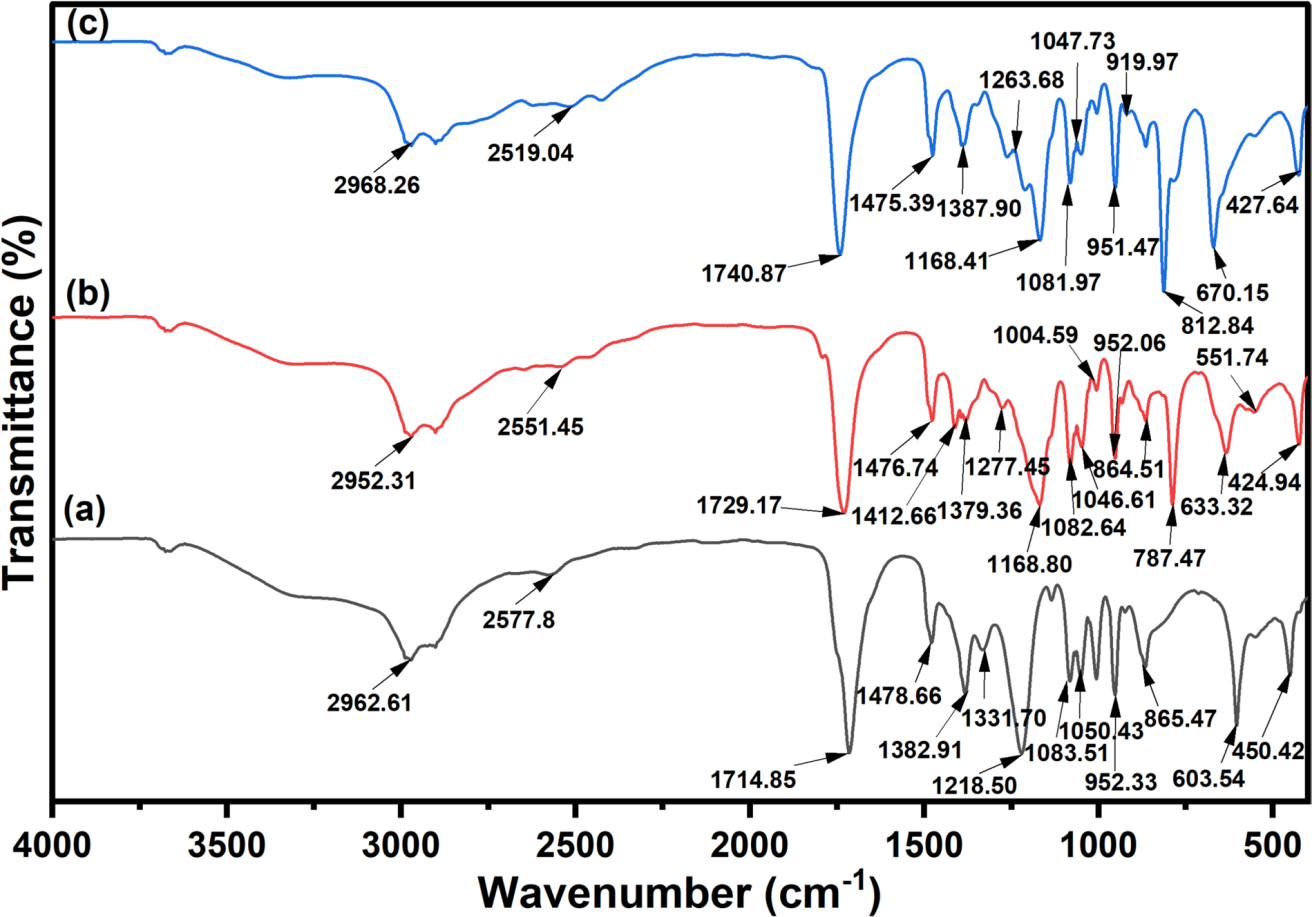
FT-IR spectra of (**a**) ChCl:AA, (**b**) ChCl:CAA, and (**c**) ChCl:DCA DESs

FT-IR analysis was used to assess the interaction that took place during the synthesis of ChCl:AA, ChCl:CAA, and ChCl:DCA DESs, as depicted in Fig. 3. The spectra were recorded over a range of 400 to 4000 cm^−1^ at room temperature. The FT-IR spectra for the synthesised DESs confirm the presence of key functional groups from both the HBA and HBDs, with distinct peaks indicating the successful formation of the DESs. For ChCl:AA DES, peaks at 2962.6 cm^−1^, 2577.8 cm^−1^, and 1714.9 cm^−1^ indicate C–H stretching in aliphatic groups, O–H stretching due to hydrogen bonding, and C = O stretching from carboxyl groups, respectively. Additional peaks between 1478.7 and 450.4 cm^−1^ correspond to various bending vibrations, C–N and C–O stretches, and skeletal vibrations, confirming ChCl and AA’s presence. In the ChCl:CAA DES, the main peaks include 2952.3 cm^−1^ for C–H stretching, 2551.5 cm^−1^ for O–H stretching in hydrogen-bonded carboxyl groups, and 1729.2 cm^−1^ for C = O stretching. C–Cl vibrations from CAA and bending and stretching vibrations involving ChCl are also evident. Similarly, the ChCl:DCA DES shows characteristic peaks at 2968.3 cm^−1^, 2519.0 cm^−1^, and 1740.9 cm^−1^ for C–H, O–H, and C = O stretching. C–Cl bending vibrations confirm the presence of chlorine in DCA. Lower-frequency peaks across all DESs are attributed to skeletal ionic interactions between ChCl and the respective HBDs. In other words, it should be noted that in carboxylic acids, the O–H stretch is typically found around 3200–3550 cm^−1^, but when hydrogen bonding occurs, the O–H stretch may shift to lower frequencies due to the strong interactions with Cl⁻ (Silverstein et al. 2005; Nakamoto 2008).

### Thermogravimetric analysis

**Fig. 4:**
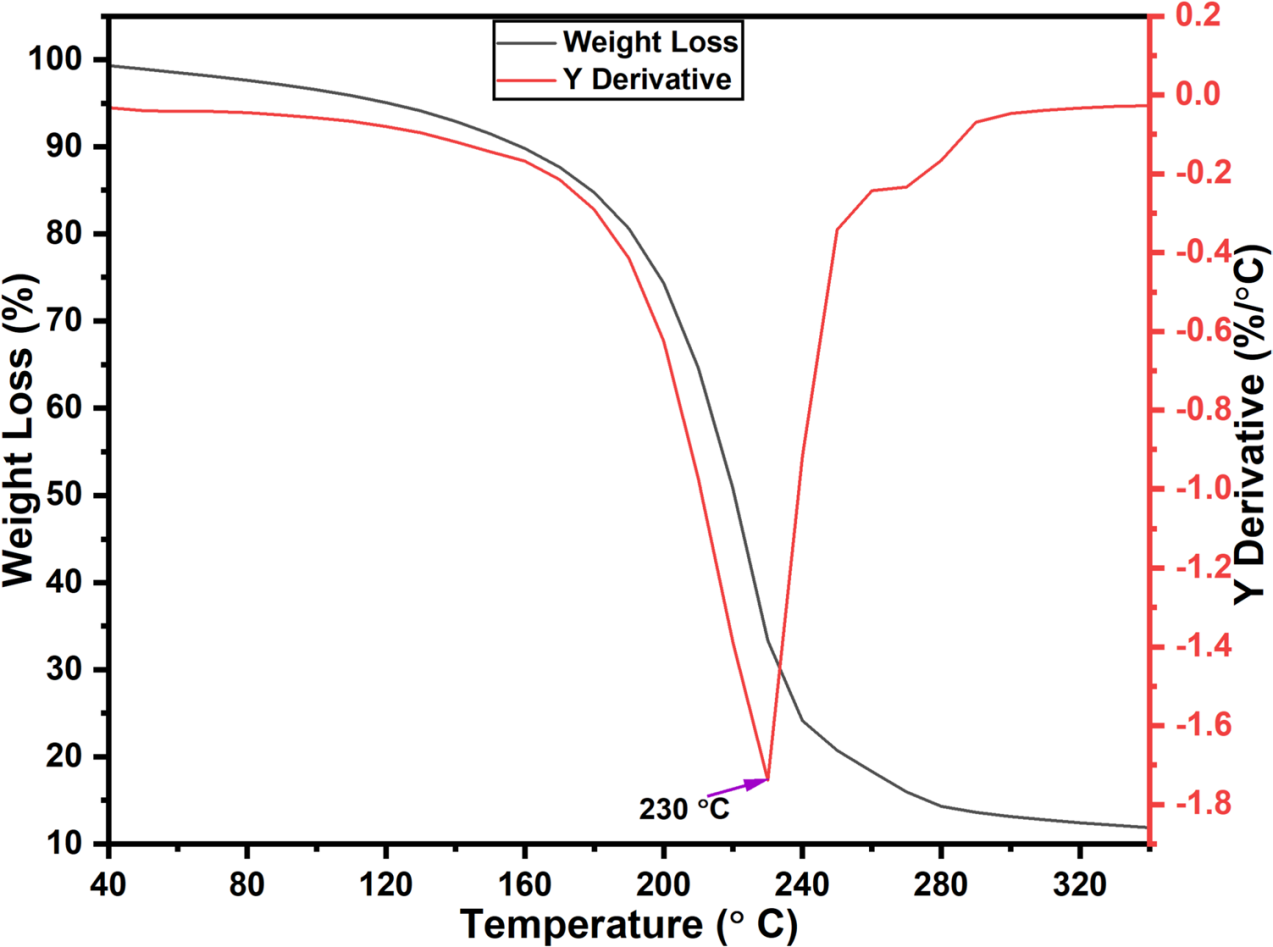
TGA profile of ChCl:DCA DES

Isothermal TGA has been employed to determine the thermal limits for the use of DESs. The thermal stability of both the individual DES components and the complete mixture is heavily impacted by the temperature sensitivity of hydrogen bonding, which plays a crucial role in determining their suitability for different applications (Zhang et al. 2024a). The initiation decomposition temperature is a critical property, as it determines the highest temperature at which DESs can retain their liquid state without decomposing (Vorobyova et al. 2024). This property subsequently defines the range of potential applications for DESs as solvents. From the thermogravimetric curves obtained, the mass loss of the substance during heating to elevated temperatures was measured (Fig. 4). In this case, a 10.0% initial mass loss, attributed to the evaporation of residual water or other volatile components, was observed between 40 and 230 °C. As the temperature continues to increase, a significant decomposition phase occurs within the temperature range of 230 to 270 °C, reaching its peak decomposition temperature at 230 °C with an initial mass loss of approximately 70%. Therefore, the operating temperature for the ChCl:DCA DES should be maintained below 230 °C to ensure thermal stability. The high thermal stability observed can be ascribed to the ionic interactions present within the DES, which create a thermodynamically advantageous liquid state (Pinho et al. 2021).

### Effects of influencing parameters

To provide context for the leaching performance results, key physicochemical properties of the DESs were considered. The density values at 298.15 K and 0.1 MPa were recorded as 1112.45 kg/m^3^ for ChCl:AA (Sheikh et al. 2024), 1278.6 kg/m^3^ for ChCl:CAA (Cui et al. 2017), and 1391.7 kg/m^3^ for ChCl:DCA, with the latter determined experimentally in this study using the same method adopted by Cui et al. (2017). The viscosities of the DESs at the same temperature followed the order ChCl:AA < ChCl:CAA < ChCl:DCA, with values of 64.8 mPa·s**,** 276.6 mPa**·**s, and 347.8 mPa·s, respectively. The viscosity of ChCl:AA was experimentally measured in this study using the same method as reported in Oke and Potgieter (2025), while those for ChCl:CAA and ChCl:DCA were adopted from that study. These values reflect the growing intermolecular interactions introduced by chlorine substitution in the HBDs, which impact both fluid flow and metal solubilisation behaviour.

In the process of recovering hazardous metals from waste PCBs using DESs, several critical parameters may play a significant role in determining the efficiency of the recovery. Some of these parameters include the nature of the DES, which dictates the solvent’s ability to interact with the target metals, as well as contact time and temperature, which influence the kinetics of the reaction. The addition of water can alter the viscosity of the DES thereby enhancing the solubility and recovery behaviour of metals, while the nature of the oxidant could affect the oxidation state of the metals, impacting their recovery efficiency. Understanding the effect of these factors is essential for optimising the process and improving the recovery of metals in an environmentally friendly manner.

#### Nature of DESs

During this study, the DESs ChCl:AA, ChCl:CAA, and ChCl:DCA were used to remove Pb, Cr, Zn, and Ni from waste PCBs to evaluate their overall effectiveness. These DESs consist of acetic acid, chloroacetic acid, and dichloroacetic acid, respectively, as their HBDs. The varying acidic strengths of these DESs, resulting from the different ionisation capacities of the acids, had a notable impact on the metal recovery efficiency. The pKa value is a measure of the strength of an acid, representing the equilibrium constant for its dissociation into ions. In simpler terms, it indicates how easily an acid releases protons (H⁺) in solution. The lower the pKa value, the stronger the acid, meaning it dissociates more readily and produces more protons. Conversely, a higher pKa value indicates a weaker acid, with less tendency to release protons (Oke et al. 2024). In the context of DESs, the pKa values of the HBDs influence the acidity of the DES (Hou et al. 2018). Acids with lower pKa values contribute to stronger acidity, which can enhance the solvent’s ability to interact with analytes, while higher pKa values result in weaker acidity, potentially reducing the solvent’s effectiveness in analytes’ recovery (Kumar et al. 2021).

**Fig. 5:**
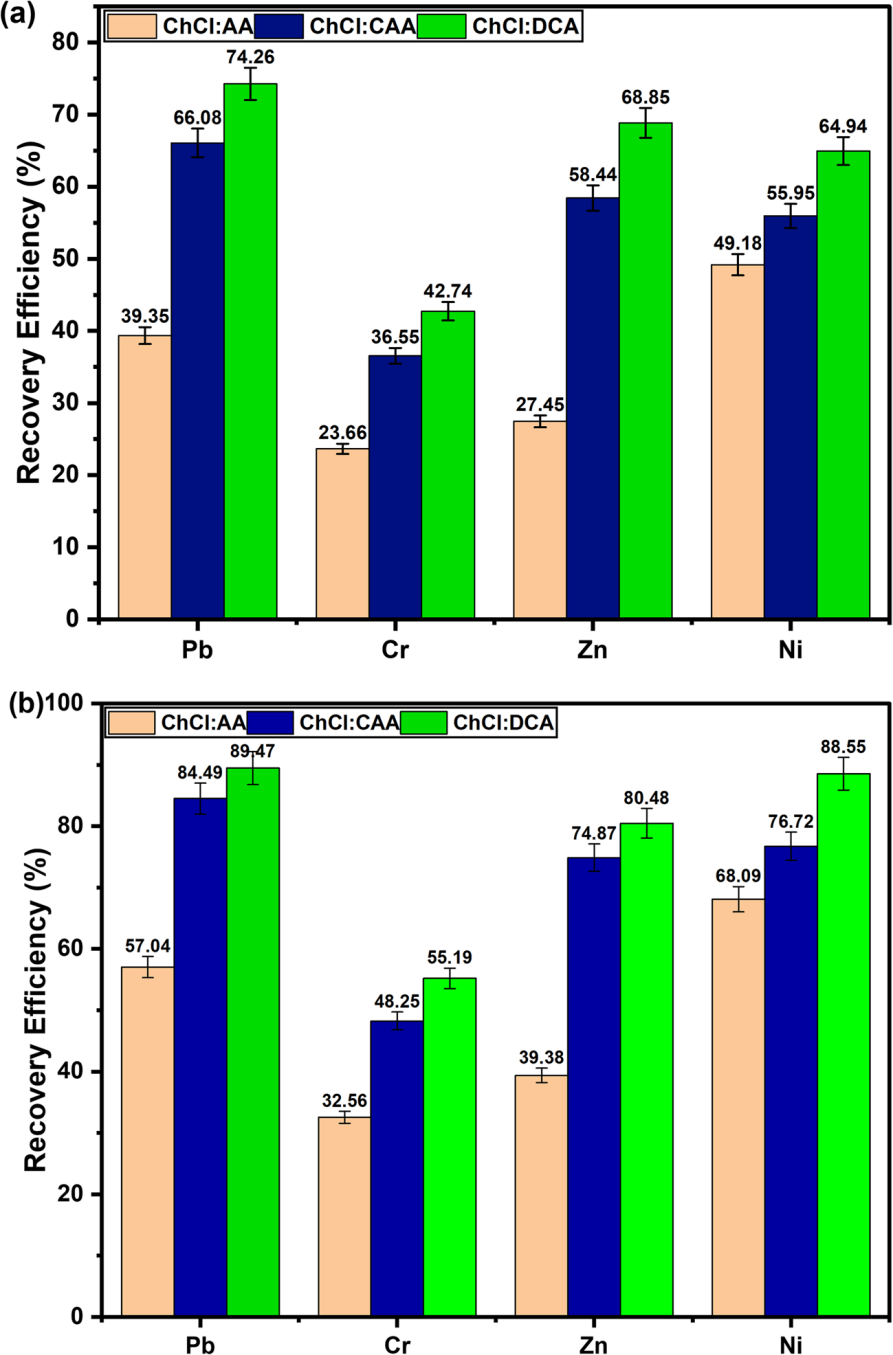
Effect of the nature of DESs on the recovery efficiency of hazardous base metals from PCBs: **a** without H_2_O_2_ and **b** with 1.0 M H_2_O_2_ (*T* = 50 °C, *t* = 3.0 h, rpm = 500, S/L = 1/10)

As illustrated in Fig. 5a and b**,** the recovery efficiency of the DESs for the studied metals improves with increasing acidity, irrespective of the presence or absence of an oxidising agent. Figure 5a presents the leaching performance of the DESs without H_2_O_2_, while Fig. 5b shows the results when 1 M H_2_O_2_ was added to the system. In both scenarios, the ChCl:AA DES consistently exhibited the lowest recovery capacities, whereas the ChCl:DCA DES demonstrated the greatest leaching efficiencies. For instance, without H_2_O_2_ (Fig. 5a), ChCl:AA achieved recovery efficiencies of 39.35% for Pb, 23.66% for Cr, 27.45% for Zn, and 49.18% for Ni, while ChCl:DCA recorded 74.26%, 42.74%, 68.85%, and 64.94% for the same metals, respectively. Upon addition of H_2_O_2_ (Fig. 5b), the recovery efficiencies increased significantly across all DESs: ChCl:AA reached 57.1% (Pb), 32.6% (Cr), 39.4% (Zn), and 68.1% (Ni), while ChCl:DCA rose to 89.5%, 55.2%, 80.5%, and 88.6%, respectively. This consistent trend confirms that the acidity of the DESs, governed by the nature of their HBDs**,** plays a critical role in metal leaching. The variation in performance is attributed to the differing pKa values of the HBDs, i.e. 4.76 for AA, 2.87 for CAA, and 1.29 for DCA, which reflects their ionisation strengths (David 2004). Thus, the increasing order of acid strength (AA < CAA < DCA) aligns with the observed enhancement in metal recovery. Moreover, the presence of electronegative chlorine atoms in CAA and DCA enhances their acid dissociation, thereby improving their capacity to dissolve metals. While the inclusion of H_2_O_2_ clearly enhances recovery efficiencies by promoting oxidative dissolution, it is evident that the intrinsic acidity of the DES remains the primary determinant of leaching performance. Consequently, ChCl:DCA DES which demonstrated the highest acidity and best performance was selected for subsequent experiments.

It is important to note that the observed recovery trends are attributed to the intrinsic properties of the DESs rather than the performance of their individual constituents. The comparison between DESs and their respective HBD or HBA components is not scientifically appropriate in this context, as DESs are non-ideal systems with undetermined molarities and emergent properties arising from strong hydrogen-bonding interactions. Additionally, choline chloride alone does not exhibit leaching capability, and the direct use of free acids at known concentrations would require an entirely different methodological framework. The aim of this work is to evaluate the efficacy of DESs as environmentally friendly lixiviants, not to benchmark their performance against conventional acids. Hence, no further experiments were conducted using the individual components in isolation.

#### Contact time

**Fig. 6:**
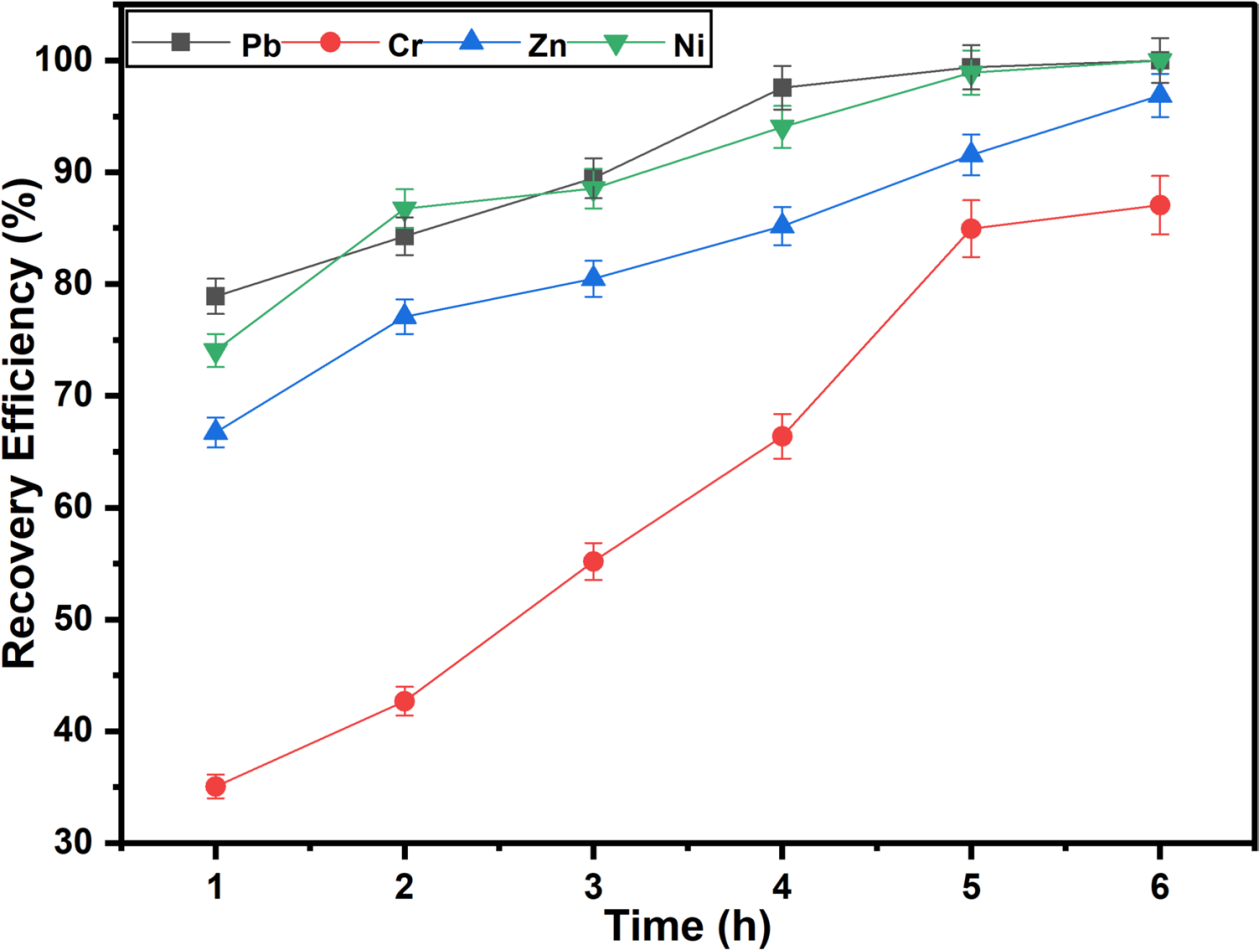
Effect of contact time on the recovery efficiency of hazardous base metals (*T* = 50 °C; *t* = 1 − 6 h; H_2_O_2_ = 1.0 M; rpm = 500; S/L = 1/10)

Since longer solvent-sample contact times allow for more thorough recovery of hazardous metals from the complex e-waste, longer solvent-PCB contact times generally improve recovery efficiency (Oke and Potgieter 2024b). In order to study its impact on metal recovery, the contact time was adjusted from 1.0 to 6.0 h as illustrated in Fig. 6. The recovery of Pb, Cr, Zn, and Ni was approximately 78.9%, 35.1%, 66.7% and 74.1%, respectively, at 1.0 h of contact time. The metal recovery increases with the contact, and the complete recovery of Pb and Ni was attained at 6.0 h of contact time. However, only 87.1% and 96.9% recovery efficiencies were respectively actualised for Cr and Zn at 6.0-h contact time. This is due to prolonged interaction between the leaching solvents and the metals, allowing more time for desorption and diffusion of metal ions from the PCB sample into the solvent. Additionally, time allows for the gradual completion of complexation reactions and dissolution processes, leading to higher extraction efficiencies (Oke and Potgieter 2024b; Oke et al. 2024).

#### Temperature

**Fig. 7:**
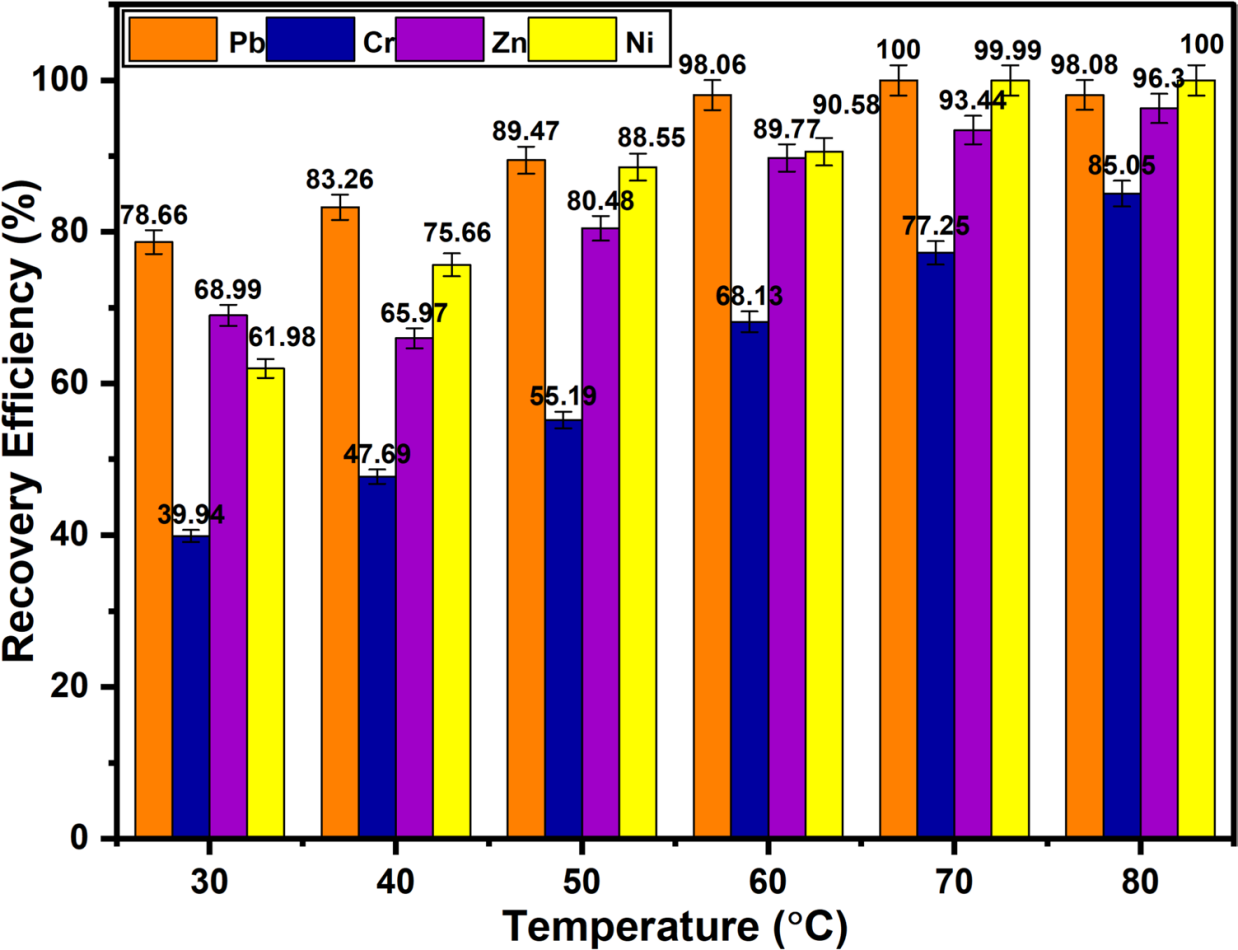
Effect of temperature on the recovery efficiency of hazardous base metals (*T* = 30 − 80 °C; *t* = 3 h; H_2_O_2_ = 1.0 M; rpm = 500; S/L = 1/10)

To assess the influence of temperature changes on the recovery of metal from waste PCBs, the temperature was adjusted between 30.0 and 80 °C. As shown in Fig. 7, the maximum recovery of Pb, Cr, Zn, and Ni at 30.0 °C was about 78.7%, 39.9%, 67.0%, and 62.0%, respectively. The leaching of Pb and Ni was complete at 70.0 °C and 80 °C, respectively. At 80 °C, however, the maximum recovery of Zn and Cr was only 96.3% and 85.1%, respectively. The enhanced reaction kinetics at higher temperatures account for the higher metal recovery at higher temperatures (Gómez et al. 2023; Zhang et al. 2024b). Higher temperatures cause molecules to collide more frequently thereby overcoming the activation energy, which raises the reaction rate and increases the recovery of metals (Oke et al. 2024; Oke and Potgieter 2025). Also, at high temperatures, the viscosity of the DESs decreases, thereby improving and enhancing the mass transfer of the metals from the sample into the DES (Binnemans and Jones 2023).

#### Addition of water

**Fig. 8:**
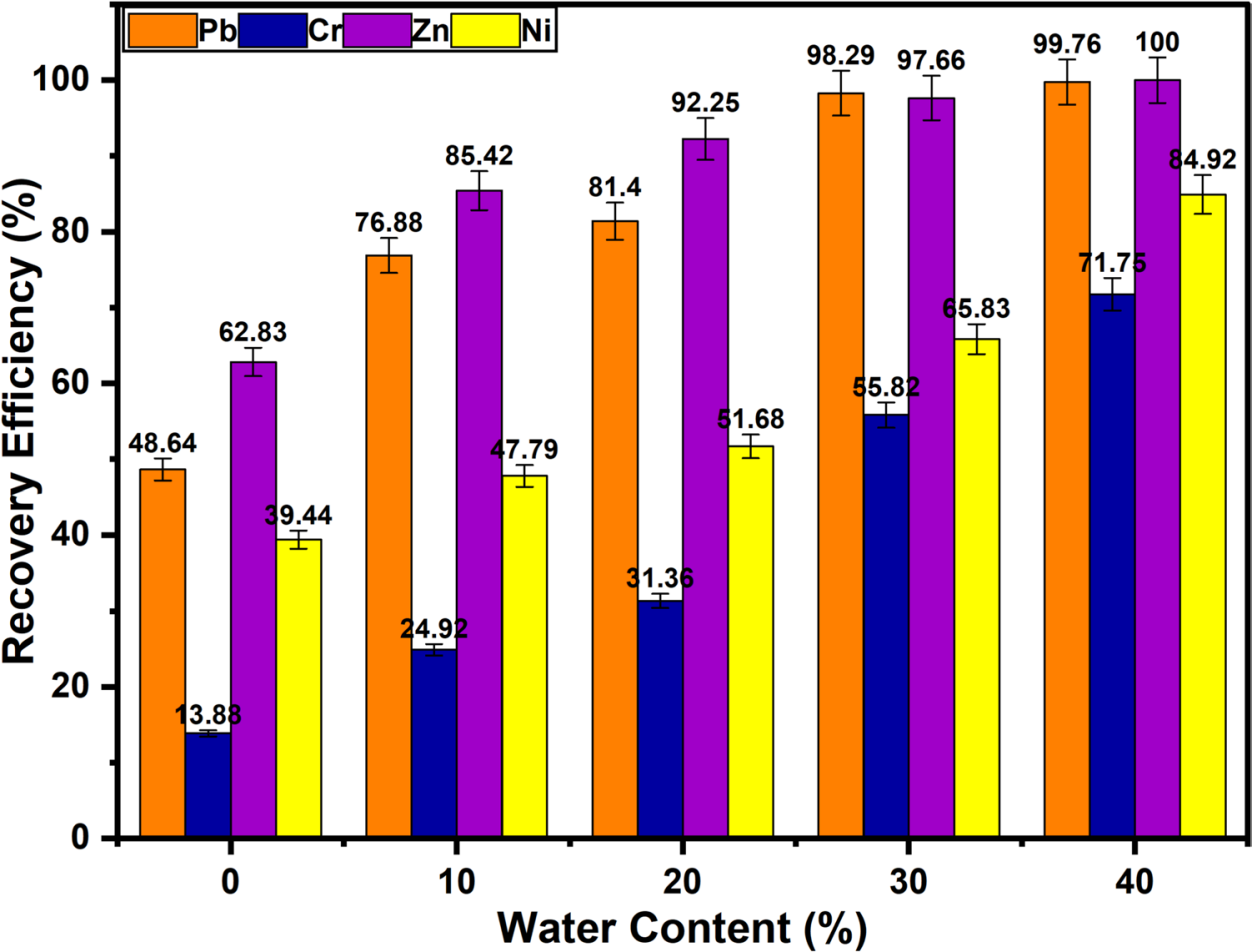
Effect of addition of water to DES on the recovery efficiency of hazardous base metals (*T* = 50 °C; *t* = 3 h; H_2_O = 0 − 40%; rpm = 500; S/L = 1/10)

The larger molecular weight of the components in DESs causes their viscosity to increase with decreasing water content, impeding the leaching process. DESs become less viscous when there is greater water content, but this also lowers the proton concentration, which makes leaching more difficult (Tang et al. 2022). Up until a water content of 42.0 wt%, most DESs maintain their usual hydrogen bond network. The mixture will therefore behave like a typical DES and not like a diluted aqueous solution if the water content is less than 42.0 wt% (Hammond et al. 2019; Zhang et al. 2024a). Based on this established threshold, the upper water content limit for this study was deliberately set at 40 wt% to ensure the DES remained unequivocally within its characteristic operational regime, maintaining its hydrogen bond network and avoiding transitional behaviour near the critical threshold. To investigate the influence of water content on recovery efficiency, a series of experiments were conducted in which the water content of the DES was systematically varied between 0 and 40%. The results presented in Fig. 8 indicate that, within the range of water content investigated in this study, the recovery efficiency of the DES improves as the water content increases. At 0% water content, the recovery efficiencies for Pb, Cr, Zn, and Ni were 48.6%, 13.9%, 62.8%, and 39.4%, respectively. However, when the water content was increased to 30% and 40%, a marked improvement in recovery efficiencies was observed for all the metals. For instance, recovery efficiencies of 99.8%, 71.8%, 100.0%, and 84.9% were achieved for Pb, Cr, Zn, and Ni, respectively, when 40% water was added to the DES. The exceptional recovery efficiencies achieved at 40 wt% water content (e.g. Pb, 99.8%; Zn, 100.0%) demonstrate that this level was sufficient to achieve near-maximal performance enhancement through viscosity reduction, without approaching the critical threshold where DES properties might be compromised. This enhanced performance is likely due to the reduction in the viscosity and an increase in the ionic mobility of the DES with increasing water content (Martín et al. 2023). So, this facilitates better interaction between the waste PCB sample and the DES, thereby improving the solvent’s capacity to extract metals from the sample matrix.

These findings are further supported by Liu et al. recently, who demonstrated that the viscosity of ChCl-based DESs decreases nonlinearly with temperature and monotonically with increasing water content (Liu et al. 2025). For example, the viscosity of pure ChCl and ethylene glycol DES was reported to drop from 47.9 mPa·s at 283.15 K to 10.1 mPa·s at 363.15 K, with additional viscosity reduction observed at hydration levels between 3 and 50 wt%. This reduction in viscosity, attributed to water’s lower intrinsic viscosity, enhances ionic mobility and diffusion within the solvent matrix. Therefore, in our system, the improved recovery efficiencies observed with increasing water content can be largely explained by enhanced mass transport and better interaction between the DES and the PCB particles, without compromising the DES’s structural integrity up to 40 wt%.

#### Nature of oxidants

**Fig. 9:**
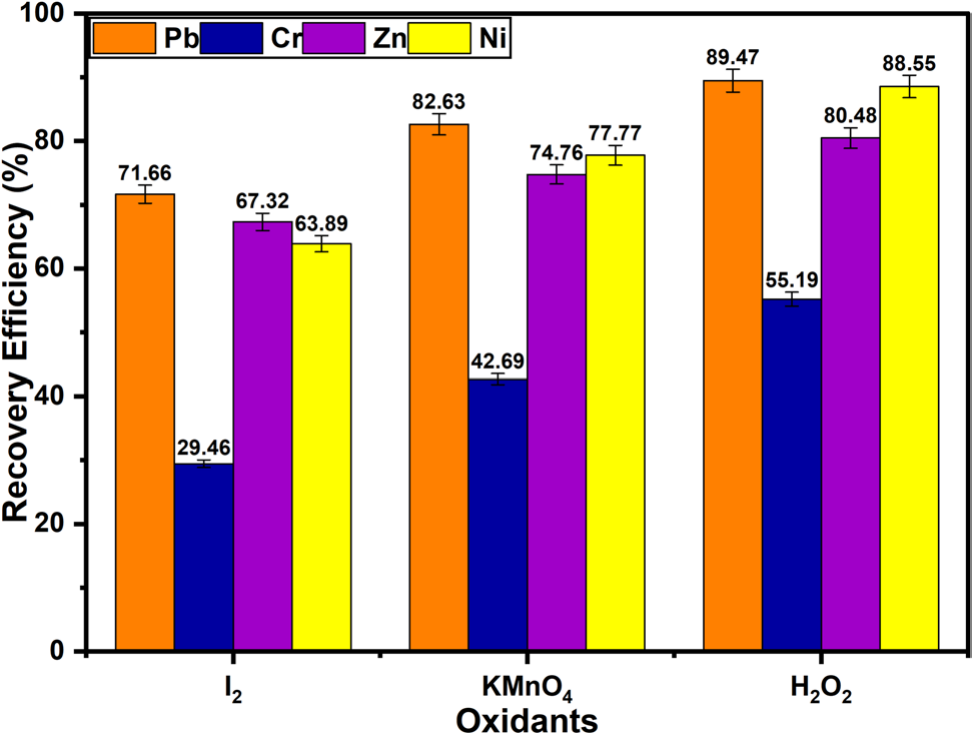
Effect of oxidant nature on the recovery efficiency of hazardous base metals (*T* = 50 °C; *t* = 3 h; H_2_O_2_ = 1.0 M; KMnO_4_ = 1.0 M; I_2_ = 1.0 M; rpm = 500; S/L = 1/10)

Oxidants play a crucial role in metal leaching processes by promoting the oxidation of metals on the surface of the sample matrix, enabling their dissolution into leaching agents. Oxidants such as H_2_O_2_, I_2_, and KMnO_4_ among others can enhance the dissolution process by accelerating the oxidation of metals in their metallic state (Perea et al. 2021). The choice of oxidant influences both the efficiency and the rate of metal extraction, making it a critical factor in the success of leaching systems. By enhancing reaction kinetics, oxidants facilitate the recovery of metals from PCBs, which is vital for optimising large-scale recycling operations (Oke and Potgieter 2024b). Therefore, we investigated the influence of 1.0 M each of H_2_O_2_, I_2_, and KMnO_4_ oxidants in the recovery of metals of interest from waste PCBs using ChCl:DCA DES for a duration of 3 h. As displayed in Fig. 9, the system containing H_2_O_2_ demonstrated the highest recovery efficiency, likely due to its strong oxidative properties and ability to generate reactive oxygen species such as hydroxyl radicals. These radicals rapidly oxidise metals in their metallic state, making them more readily soluble in the ChCl:DCA DES. The small size and high reactivity of these radicals ensure efficient penetration into the PCB matrix, leading to superior metal extraction. In contrast, the lowest recovery efficiency was observed in the system using I_2_ as the oxidant. I_2_ has a relatively lower oxidative potential and slower reaction kinetics compared to H_2_O_2_. Although it can oxidise metals, its weaker and more selective oxidation process is less effective at promoting the dissolution of metals in their metallic form, which explains the reduced recovery efficiency (Oke and Potgieter 2024b). KMnO_4_, though more effective than I_2_, exhibited lower recovery efficiency compared to H_2_O_2_ due to its different oxidation mechanism. While KMnO_4_ is a strong oxidant, its larger molecular structure and slower diffusion rate in the DES limits its interaction with the metallic surfaces of the PCBs, resulting in less efficient metal extraction. A similar observation was reported by Perea et al. in 2021 during Cu recovery, where the system consisting of H_2_O_2_ consistently exhibited a higher Cu dissolution rate compared to KMnO_4_ (Perea et al. 2021). In summary, the ability of the different oxidants studied towards the recovery of the examined metals follows the sequence of H_2_O_2_ > KMnO_4_ > I_2_.

### Analyses of the unleached and leached waste PCBs

**Fig. 10:**
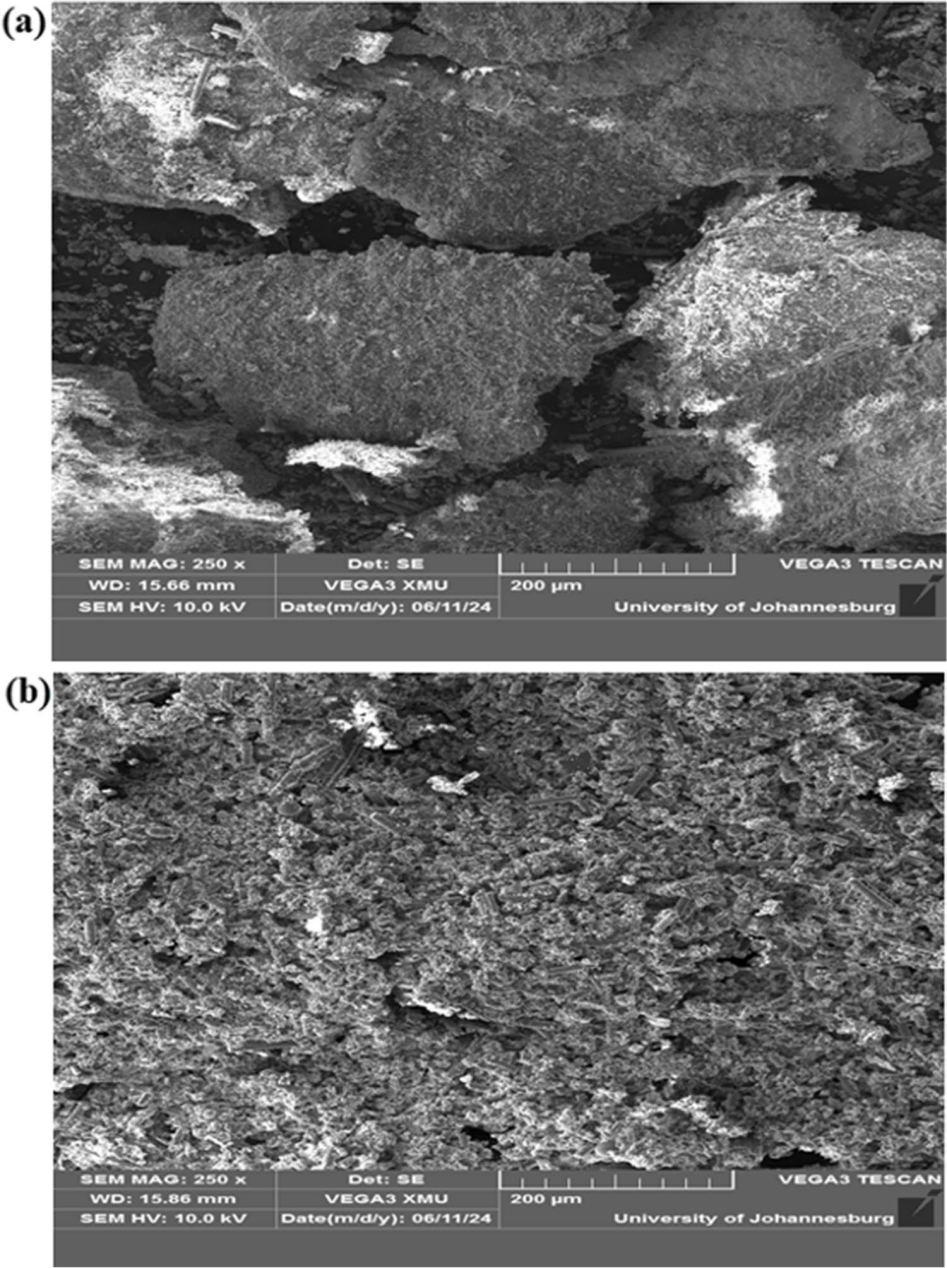
SEM images of **a** unleached waste PCBs and **b** leached waste PCBs

The SEM images in Fig. 10a and b, alongside the XRF elemental analysis (Table 3), provide comprehensive evidence of the morphological and compositional transformations in waste PCBs following the leaching process using ChCl:DCA DES. In Fig. 10a, the unleached (pre-leached) PCB sample exhibits a dense and rough surface with large, irregular fragments. Block-like structures are visible, indicating the presence of intact metallic particles or agglomerates. The surface is further characterised by cracks and voids, reflecting the heterogeneous and unprocessed nature of the PCB material, which typically consists of metals and other components. The minimal surface degradation observed suggests that the material remained largely unaltered prior to leaching. Similar observations were reported by Chu et al. in their recent work involving the SEM analysis of waste PCBs (Chu et al. 2022). Conversely, Fig. 10b, representing the leached PCB sample, shows significant morphological changes. The previously large, blocky fragments have been broken down into smaller, finer particles, resulting in a more porous and granular surface. The disintegration of the structures seen in Fig. 10a indicates that the DES effectively dissolved and extracted the metallic constituents. The increased porosity and reduced structural integrity demonstrate the efficiency of the leaching process, leaving behind a brittle, more homogeneously distributed residue. This visual evidence is further corroborated by the XRF elemental analysis (Table 3), which confirms the quantitative transformation of the residual product. The XRF data highlights a marked reduction in the metal content of the leached sample, supporting the observations made in Fig. 10b. The structural changes observed in the SEM images align with the compositional alterations shown by the XRF analysis, providing strong evidence of successful metal extraction. Together, these findings demonstrate the effectiveness of the ChCl:DCA DES in breaking down the PCB material and leaching the target metals, transforming the PCB from a dense, compact form to a more porous and structurally degraded state.

**Table 3:** XRF results of hazardous metals in waste PCBs sample after the recovery process

Ni (mass %)	Zn (mass %)	Pb (mass %)	Cr (mass %)
0.025	0.011	0.076	0.016

### Comparison with the literature

The comparison of recovery efficiencies of various recovery methods for hazardous base metals from waste PCBs is presented in Table 4. Methods such as bioleaching have demonstrated high efficiency for metals like Ni and Zn; however, this method is slower and can yield variable results based on the specific metal and conditions used. Alkali fusion has also shown significant recovery efficiencies for Pb and Zn, but it involves complex processes. The electrochemical hydrochlorination method provides variable efficiencies for Pb, Cr, Zn, and Ni, indicating its potential for certain metals while also highlighting its limitations for others. In contrast, DES from this study showed remarkable recovery efficiencies for Pb, Cr, Zn, and Ni, underscoring the effectiveness of these newly developed methods.

**Table 4:** Comparison of our results with available methods

Methods	Techniques	Metals	Efficiencies (%)	References
Bioleaching	AAS	Ni Zn	99.8 98.0	Shah et al. (2015)
Bioleaching	ICP-OES	Pb Cr Ni Zn	10.0 75.0 90.7 90.8	Gu et al. (2019)
Alkali fusion	ICP-AES	Pb Zn	79.0 91.0	Guo et al. (2015)
Alkaline glycine	AAS	Pb Zn Ni	46.8 92.5 12.6	Li et al. (2020)
Electrochemical hydrochlorination	ICP-OES	Pb Cr Zn Ni	5.1 84.4 100.0 100.0	Serga et al. (2023)
ChCl:DCA DES	ICP-MS	Pb Cr Zn Ni	99.8 71.8 100.0 84.9	This study

### Kinetic study

The recovery temperatures and times were varied from 30 to 70 °C and 0.5 h and 3 h, respectively, while other parameters were kept constant. Recovery kinetics can be used to examine the interaction between unreacted particles and the recovery agent, enabling the determination of the limiting steps and the reaction’s activation energy. Metals dissolve in the ChCl:DCA DES recovery platform through a solid–liquid multi-phase reaction that takes place at the interface between the two phases. In other words, the reaction for metal recovery in the current study begins at the particle’s outer surface, causing the reaction zone to shrink as the reaction time progresses. Because of this, the reaction zone shifts inward, leaving the removed metals in the aqueous solution. Owing to this, the most popular kinetic model for this kind of reaction called the shrinking core model (SCM) was employed. The chemical reaction at the particle surface and diffusion are anticipated to be the rate-limiting steps. Equations 2 and 3 represent the SCM for the recovery controlled by chemical reaction and diffusion, respectively (Wang et al. 2022; Jadhao et al. 2023; Oke et al. 2024).

2 $$1-\:{(1-X)}^{1/3}=k_1t$$

3 $$1-3{(1\:-\:X)}^{2/3}\:+2(1-X)=k_2t$$

In this case, *k*_1_ and *k*_2_ = rate constants (min^−1^), *t* = time (min), and *X* = recovered fraction of metal.

**Fig. 11:**
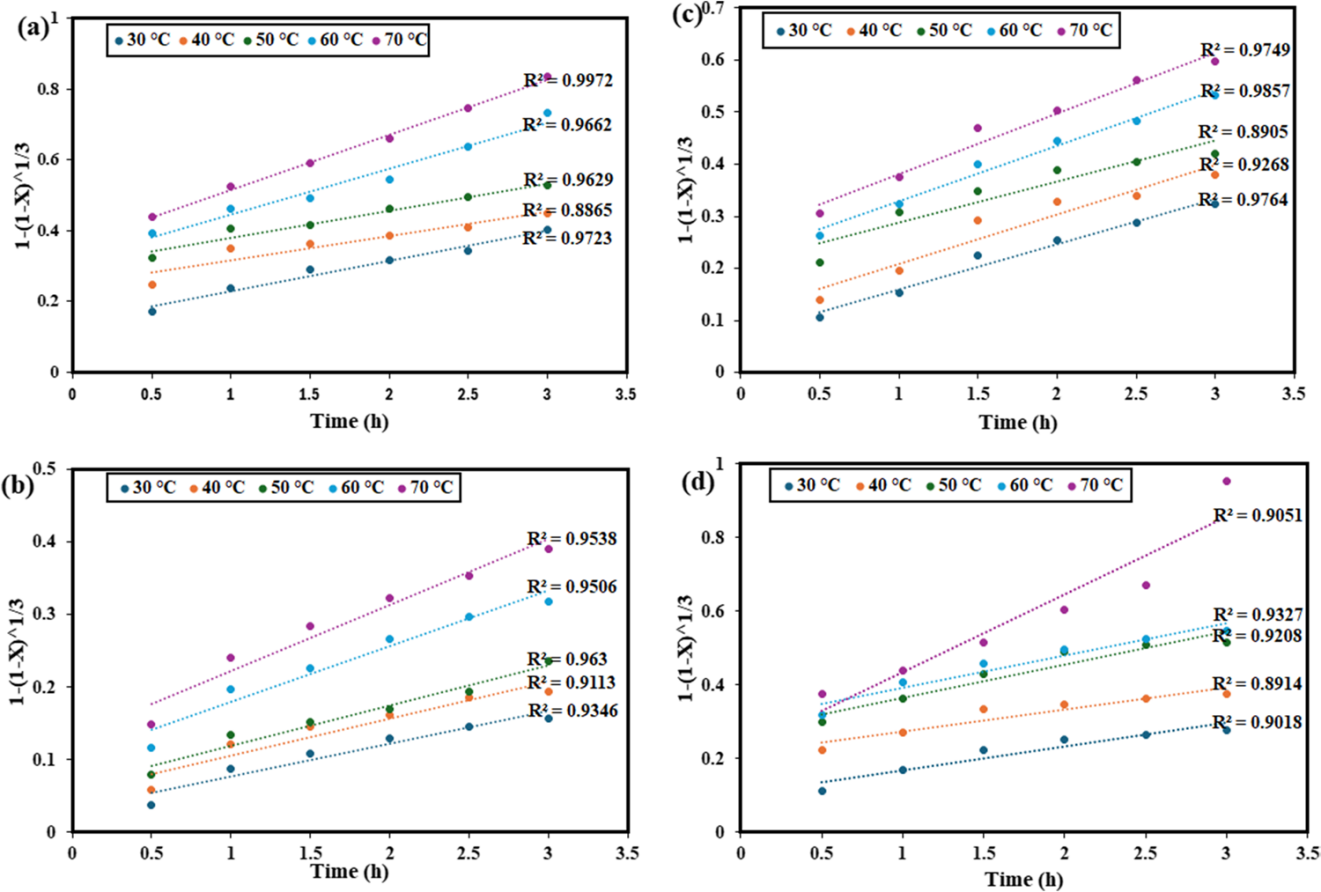
Linear fitting results of 1 − (1 − *X*)^1/3^ with various times at different temperatures by the chemical reaction control model for **a** Pb, **b** Cr, **c** Zn, and **d** Ni from waste PCBs using ChCl:DCA DES

**Fig. 12:**
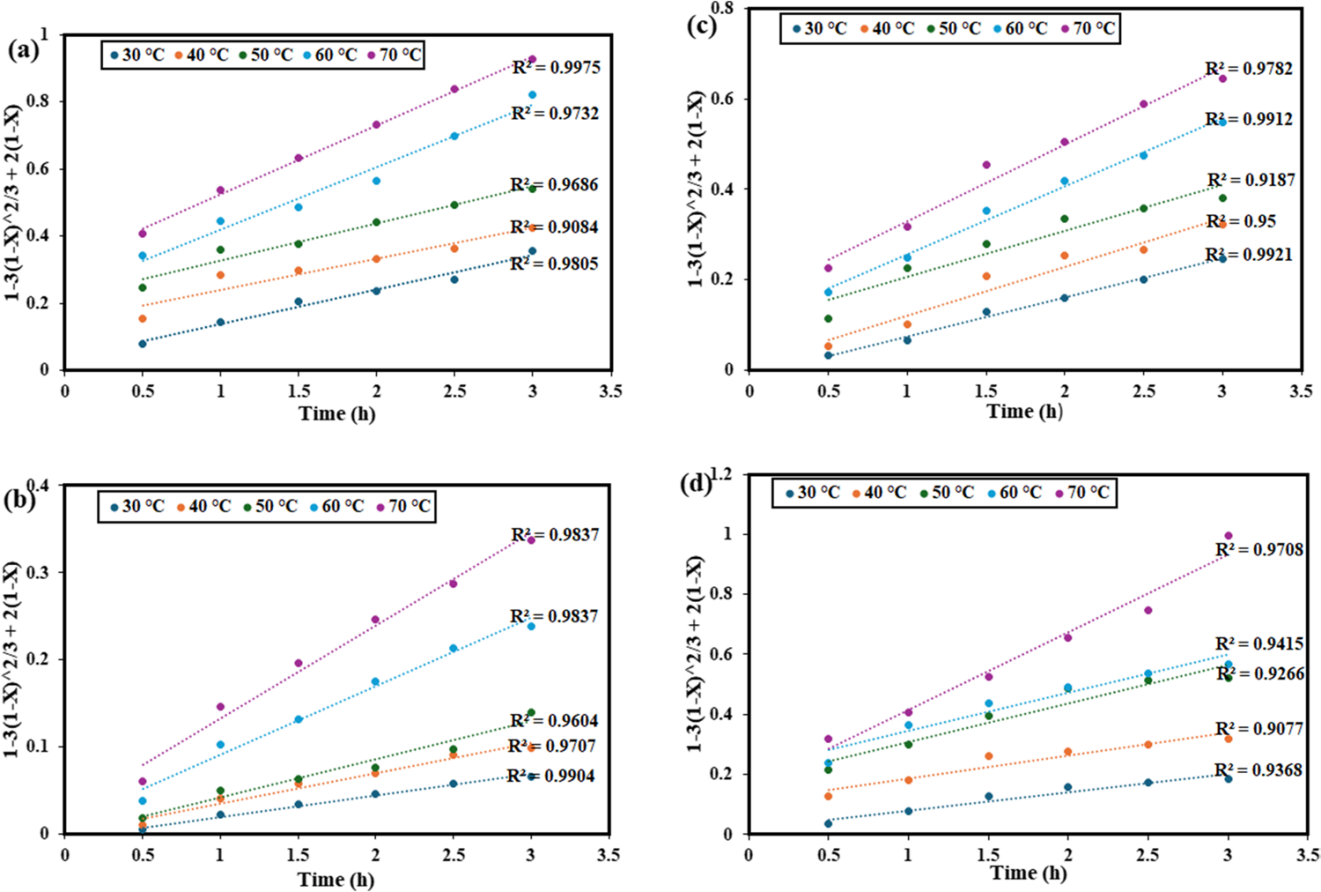
Linear fitting results of 1 − 3(1 − *X*)^2/3^ + 2(1 − *X*) = *kt* with various times at different temperatures by the chemical reaction control model for **a** Pb, **b** Cr, **c** Zn, and **d** Ni from the waste PCBs using ChCl:DCA DES

In Fig. 11, the plot of 1 − (1 − *X*)^1/3^ versus time at different temperatures shows that the data for these metals do not follow a linear trend perfectly. The low correlation coefficients (*R*^2^ values) indicate that the chemical reaction-controlled SCM is not applicable in describing the recovery process for these metals. Therefore, it can be concluded that the recovery of Pb, Cr, Zn, and Ni is not controlled by chemical reaction kinetics. In contrast, Fig. 12 shows the plot of 1 − 3(1 − *X*)^2/3^ + 2(1 − *X*) versus time, representing the diffusion-controlled model, which demonstrates a much better fit for the experimental data. The higher *R*^2^ values suggest that diffusion, rather than chemical reaction, is the rate-determining step. Additionally, the increasing slopes with temperature indicate that higher temperatures enhance the recovery efficiencies, suggesting that temperature plays a significant role in facilitating the diffusion process. Therefore, it can be inferred that the recovery of Pb, Cr, Zn, and Ni from waste PCBs using ChCl:DCA DES is governed by a diffusion-controlled mechanism, with temperature being a crucial factor in improving the efficiency of metal recovery. A similar observation was reported by Jadhao et al. in their recent work in which methanesulfonic acid was employed for the recovery of Cu, Zn, and Ni from waste PCBs (Jadhao et al. 2023).

The Arrhenius equation describes the relationship between the reaction rate constant (*k*) and temperature as shown in Eq. (4). This relationship indicates that as temperature rises, the rate constant *k* increases because the added thermal energy helps reactant molecules overcome the activation energy barrier, leading to more frequent effective collisions.

4 $$k=Ae^{-Ea/RT}$$

**Fig. 13:**
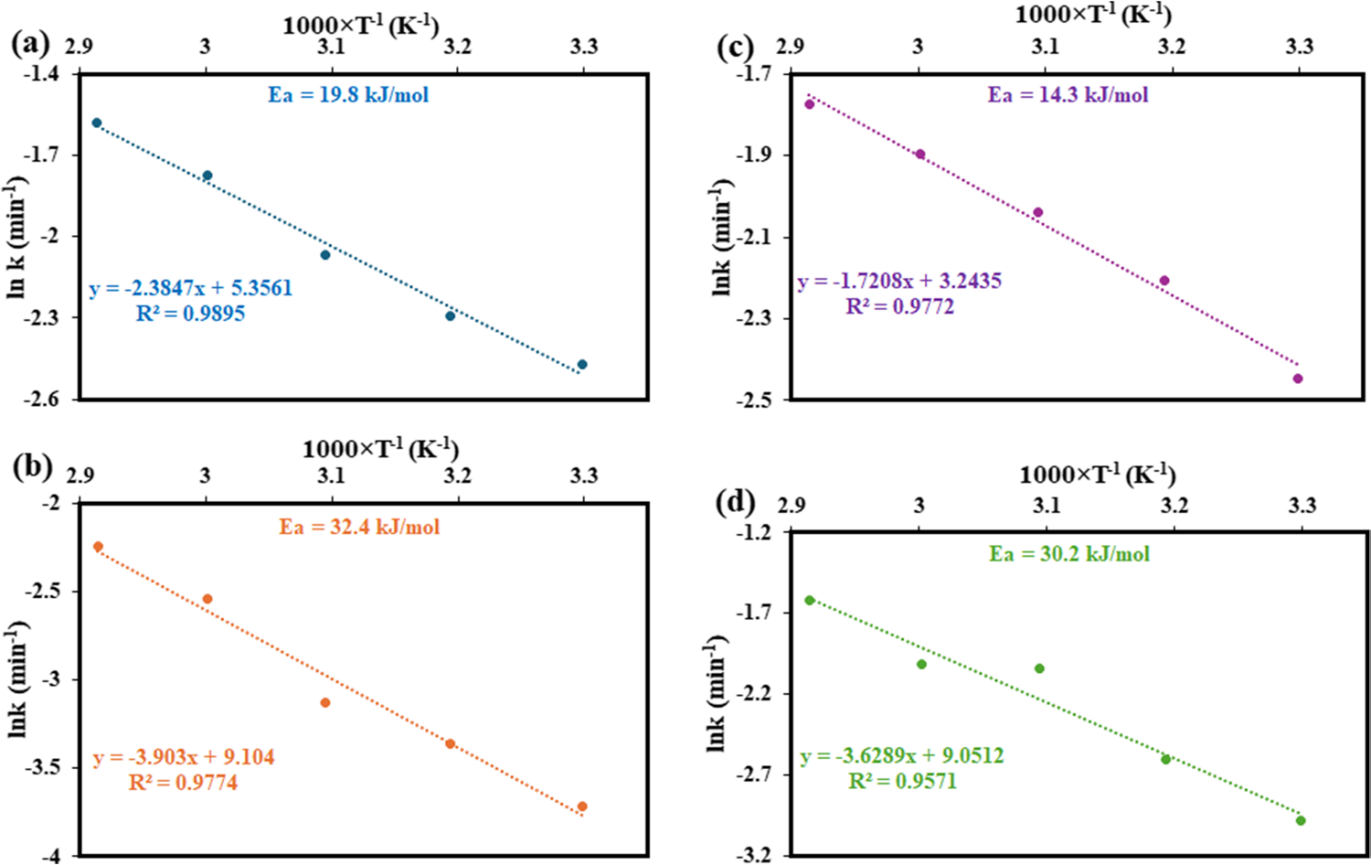
Arrhenius plots and the estimated Ea for the recovery of **a** Pb, **b** Cr, **c** Zn, and **d** Ni using ChCl:DCA DES

In this equation, *k* is the rate constant, *A* is the frequency factor, representing how often reactants collide, Ea is the activation energy, or the energy threshold for the reaction to proceed, *R* is the universal gas constant (8.314 J/mol/K), and *T* is the temperature of the reaction in Kelvin. The apparent activation energies (Ea) of the recovery reactions were determined by employing the linear form of the Arrhenius equation depicted in Eq. 5 based on the data gotten from the diffusion-controlled SCM. The plot of ln *k* versus 1/*T* for Pb, Cr, Zn, and Ni recovery is displayed in Fig. 13.

5 $$In\;k=InA-Ea/RT$$

The activation energy values obtained for the recovery of Pb, Cr, Zn, and Ni from waste PCBs using the ChCl:DCA solvent, as presented in Fig. 13, were estimated to be 19.8 kJ/mol, 32.4 kJ/mol, 14.3 kJ/mol, and 30.2 kJ/mol, respectively, over the temperature range of 30 to 70 °C. According to the literature, when the apparent activation energy is below 40 kJ/mol, the process is likely to be diffusion-controlled, whereas values above 40 kJ/mol indicate a chemically controlled rate-determining step (Teimouri et al. 2022; Olaoluwa et al. 2023; Oke et al. 2024). In our study, all activation energies for the metals investigated are under the 40 kJ/mol threshold, which implies that the recovery of Pb, Cr, Zn, and Ni using ChCl:DCA is primarily diffusion-controlled. This agrees with the conclusion obtained using SCM. This implies that the overall recovery process may be influenced more by the transport of metal ions within the solvent matrix rather than by the intrinsic chemical reactions at the solid–liquid interface. Such diffusion-controlled mechanisms often lead to enhanced reaction rates at moderate temperatures, as seen in our results. This outcome is advantageous for practical applications, as it supports the potential for efficient metal recovery under relatively mild conditions without the need for high energy inputs.

## Conclusions

This study has demonstrated the significant potential of acidic DESs for the recovery of hazardous metals from waste PCBs, providing an environmentally sustainable alternative to conventional methods. The thermogravimetric analysis confirmed that DESs exhibit high thermal stability, with the ChCl:DCA DES maintaining stability up to 230 °C, broadening their applicability in metal extraction processes requiring elevated temperatures. Among the DESs investigated, ChCl:DCA outperformed others in recovering Pb, Cr, Zn, and Ni, showing a clear correlation between the acidity of the HBDs and metal recovery efficiency. The results revealed that higher acidity, influenced by the presence of electronegative chlorine atoms, significantly enhanced the interaction with target metals. ChCl:DCA, with its strongest acidity, proved particularly effective, achieving superior recovery of these four metals compared to ChCl:AA and ChCl:CAA. Contact time was a crucial parameter, with prolonged exposure resulting in higher recovery rates, particularly for Pb and Ni, which were completely removed after 6 h. Temperature also played a key role, as elevated temperatures improved metal recovery by accelerating reaction kinetics and reducing solvent viscosity, which enhanced mass transfer for all metals. The addition of water to the DES was found to balance viscosity reduction and proton concentration, with a water content of 40 wt% delivering the highest recovery efficiencies for Pb, Cr, Zn, and Ni. The study also highlighted the significant impact of oxidants in promoting metal dissolution, with H_2_O_2_ emerging as the most effective due to its ability to generate reactive oxygen species, which rapidly oxidise metals in their metallic state, facilitating their recovery. The sequence of oxidant effectiveness for the metals followed the trend: H_2_O_2_ > KMnO_4_ > I_2_. The SCM reveals that the recovery of Pb, Cr, Zn, and Ni from waste PCBs using ChCl:DCA DES is governed by a diffusion-controlled mechanism. The activation energies were determined to be 19.8 kJ/mol for Pb, 32.4 kJ/mol for Cr, 14.3 kJ/mol for Zn, and 30.2 kJ/mol for Ni, respectively, further confirming diffusion as the rate-controlling step. Overall, this research underscores the versatility and efficacy of acidic DESs, particularly ChCl:DCA, in the sustainable recovery of Pb, Cr, Zn, and Ni from e-waste. These findings pave the way for the development of green technologies for recovering hazardous metals from waste PCBs. Also, the developed method is energy-efficient and economically viable, aligning well with goals of sustainability and environmental protection.

Although this study focused on the efficient leaching of metals from WPCBs using carboxylic acid-based DESs, the selective separation of individual metals from the resulting leachate remains a critical step for achieving high-purity metal products and industrial feasibility. Recent studies have explored various strategies, such as hydrophobic deep eutectic solvents, ionic liquids, synergistic extractants, and phase engineering approaches, to achieve this goal (Chen et al. 2024; Guo et al. 2025a, b; Zhang et al. 2025). These developments highlight the importance of coupling efficient leaching with robust separation protocols in order to advance circular economy solutions for electronic waste. Our future work will explore metal-specific separation pathways and DES recyclability in line with these developments. Also, in future work, attention will be directed towards improving the recovery, purification, and reuse of the DESs used in this study. Preliminary attempts revealed that the recycled solvents contained impurities that impaired their effectiveness, underscoring the need for more refined recycling protocols. Addressing this challenge is essential for realising a more sustainable and circular leaching process.

## Data Availability

The original contributions presented in the study are included in the manuscript. Further inquiries can be directed to the corresponding author.
